# Iodine deficiency in pregnant women at first trimester in Ankara

**DOI:** 10.4274/jtgga.galenos.2018.2017.0150

**Published:** 2019-02-26

**Authors:** Kazibe Koyuncu, Batuhan Turgay, Feride Söylemez

**Affiliations:** 1 Department of Obstetrics and Gynecology, Ankara University School of Medicine, Ankara, Turkey

**Keywords:** Iodine deficiency, pregnancy, first trimester

## Abstract

**Objective:**

Iodine deficiency in pregnant woman in Ankara was shown in previous studies. We aimed to conduct a study in a tertiary center to investigate the need for iodine replacement in our population.

**Material and Methods:**

This was a single tertiary center, non-interventional, retrospective, cross-sectional study. Data were retrieved retrospectively from 440 women who were in the first trimester in gestational age. Maternal iodine status, thyroid-stimulating hormone (TSH) levels and T4 levels were examined. Urinary iodine concentration (UIC) was calculated based on the Sandell-Kolthoff reaction, which is a colorimetric method. We excluded patients with previous or current thyroid disease. Thyroid hormones and TSH were measured using chemiluminescence immunoassays.

**Results:**

Iodine deficiency prevalence (urinary iodine <150 μg/L) was 84.7% in first trimester of pregnancy in our population. The median UIC was 81.6 (1-450) μg/L, indicating iodine insufficiency. All the patients declared iodized salt use. None of the patients were taking iodine replacement. The mean TSH level was 1.53±1.27 mIU/L, (0.01 mIU/L-14.74 mIU/L) and the mean T4 level was 12.51±5.01 mIU/L (7.09 mIU/L-23.7 mIU/L). The TSH levels of 56 patients were higher than 2.5 mIU/L. According to these results, 12.72% of the patients had subclinical hypothyroidism based on serum TSH and free thyroxine levels. Isolated hypothyroxinemia was present in one patient.

**Conclusion:**

Our study demonstrated that pregnant women still develop iodine deficiency in Ankara despite mandatory iodine salt use. Iodized salt use does not provide enough iodine supplement, especially in pregnant women. Iodine supplementation has been shown to enhance neurologic development and psychomotor performance. We suggest that iodine should be a part of routine laboratory evaluation at the first prenatal visit for its importance in early pregnancy. Also, iodized salt use education should be provided to women to eradicate iodine deficiency. Iodine supplements should be recommended to all pregnant women in addition to iodized salt.

## Introduction

Iodine deficiency is still a serious public health problem all over the world despite combative efforts (1). In previous studies, iodine deficiency status was determined by calculating urinary iodine concentration (UIC) in school age children (2). If the median results of urinary iodine levels were below 100 μg/L, it could be concluded that iodine intake is insufficient in the whole population (3). The World Health Organization (WHO) suggest classification for UIC, which is enough between 150-249 μg/L, insufficient if below 150 μg/L, and excessive above 250 μg/L (3). Iodine status is important in pregnancy because of its importance of maternal thyroid hormone production for fetal central system maturation (4). Severe iodine deficiency was found to be associated with mental retardation, decreased brain development, and low intelligence (5,6). Physiologic changes in pregnancy such as increased glomerular infiltration and the developing fetal thyroid gland increases the need for iodine beginning in the early weeks of pregnancy. The WHO recommends 250 μg/L iodine intakes for pregnant and lactating women (7). Iodine deficiency may cause diffuse or nodular goiter, hypothyroidism, and hyperthyroidism. Although there are approved treatment modalities for hypothyroidism and hyperthyroidism in pregnancy, treatment of subclinical hypothyroidism is still controversial. A recent meta-analysis showed that subclinical hypothyroidism is relevant to lower intelligence and motor scores in children (8). In some studies, subclinical hypothyroidism was reported to relate with increased risk for low birth weight, premature delivery, fetal distress, and fetal growth restriction (9,10). Subclinical hypothyroidism should be treated with 50 μg/daily levothyroxine (LT4) before conception or during gestation (11,12). Iodine status can be assessed through measurement of UIC, thyroid size, thyroglobulin, and neonatal serum thyroid-stimulating hormone (TSH) (13,14). UIC indicates current iodine nutrition, on the other hand, other methods reflect long-term iodine status. Iodination of salt is the first choice for iodine replacement (15,16). After recognizing iodine deficiency from tudies in Turkey, the Ministry of Health has obliged companies to iodinate table salt and sell it in proper storage since 1994. It has been shown that a decade of mandatory iodine prophylaxis was enough to eradicate goiter among school children (16). Ankara was shown to be an iodine-sufficient region of Turkey (median UIC in school age children; 135 μg/L), after mandatory iodization of salt (16). Herein, we aimed to show the iodine status in pregnant women in Ankara and to clarify the need for iodine replacement with iodine supplementation.

## Material and Methods

The present study was conducted in a tertiary center, between January and July 2016. Four hunded sixty women who presented to hospital for the first visit of pregnancy were retrospectively analyzed. The mean age of the pregnant women was 27.8±5.71 years. The median gestational age of the patients was 7 weeks (6-10 weeks). Patients were evaluated for fasting blood and urine samples in this routine first trimester visit. TSH, free triiodothyronine (T3), and free thyroxine (FT4) levels were assessed from the serum. Immunochemiluminescent assays performed on an automated analyzer (Advia Centaur XP; Siemens) were used to measure levels of TSH, free T3, and FT4. The UIC was determined using a colorimetric method based on the Sandell-Kolthoff reaction as recommended by the WHO and the International Council for Control of Iodine Deficiency Disorders, using Fisher reagents (17). The analytical sensitivity was 2 μg/L. The coefficient of variation was <5% for the measurement range. A single-spot urine analysis was taken from the patients in the morning (between 09.00 and 12.00 hours) and stored in de-iodized I tubes at -40 °C. UIC was measured using the spectrophotometric method described by Sandell-Kolthoff. The coefficient of variation in the range investigated was <5%. Normal serum T3 and T4 values for nonpregnant women were 3.99-6.71 pmol/L and 7-15.96 pmol/L, respectively. The results showed inter- and intra-assay coefficients of variation <5% for the measurement range. Ensuring the Quality of Iodine Procedures and Center for Disease Control programme have been performed quarterly in our laboratuary since 2000. Laboratory external control reports have shown a success rate of 85-90%. Patients were excluded if they were on thyroid medication or were known to have thyroid diseases such as thyroiditis or hypo-hyperthyroidism. Twenty patients were excluded due to these criteria, so the study was concluded with 440 patients.

### Statistical analysis

Data were analyzed using the Statistical Package for the Social Sciences software [SPSS, version 15.0; standard deviation or, if not normally distributed, as medians (ranges)]. Statistical analysis was performed using parametric (v2 - and Student’s t-tests) or nonparametric (Fisher’s exact and Mann-Whitney U) tests, when appropriate. Values for p<0.05 were accepted as statistically significant.

### Ethics

The study was approved by the Ethics committee of Ankara University School of Medicine (approval no: 12-568-16).

## Results

Four hundred forty pregnant women whose ages were between 17 and 45 (27.86±5.71) years were enrolled for the study before 12 weeks of gestation. The median gestational age was seven weeks. We found that the median UIC was 81.6 μg/L (1-414 μg/L) in pregnant women, which was described as insufficient iodine intake according to the WHO criteria. UIC was below 150 μg/L in 373 women (86.7%); 9 (2.04%) women had UIC above than 250 μg/L, and only 58 women (13.24%) had adequate iodine intake. UIC in the study group is shown in Table 1. UIC were below 50 μg/L in 149 patients (33.6%). None of the patients had UIC levels higher than 500 μg/L. The prevalence of iodized salt consumption was 100%. Of the 440 patients, 56 (12.72%) patients’ TSH levels were found to be higher than 2.5 mIU/L with normal FT4, which was diagnosed as subclinical hypothyroidism. One patient (0.22%) had isolated hypothyroxinemia with low FT4 (<7 pmol/L) and normal TSH concentrations. Thyroid values are shown in Table 2. We found no significant correlation between TSH and urinary iodine. (Spearman’s correlation coefficient r=-0.009, p=0.874). Patients with subclinical hypothyroidism were treated with 50 mcg LT4. Isolated hypothyroxinemia was treated with only iodine replacement. Also, patients with iodine deficiency were given iodine replacement.

## Discussion

Iodine deficiency is still a public health problem in Turkey (18). Despite the provision of mandatory iodinized salt, studies showed mild-to-severe iodine deficiency in the pregnant population (19). UIC levels have been accepted as a good indicator for assessing iodine status (18). Although studies in school age children showed enough iodine intake after mandatory iodized salt, it does not precisely represent iodine status in pregnant women (20). In our study, we found that iodine deficiency was still high in the pregnant population. The median UIC levels were found as 81.67 μg/L, and were below 50 in 33.6% of patients, and this was in a city that was found to be iodine sufficient in previous studies (19). Iodine deficiency is known to have an adverse effect on fetal development (20). Iodine is the essential component of T4 and T3. Proper iodine replacement is necessary for appropriate development of the fetus (3). Thyroid hormone is essential for normal maturation of the central nervous system (21). The fetus is completely dependent on maternal T4 during the first trimester of pregnancy. The production of TSH by the fetal pituitary gland start from week 18-22 of gestation, the production of fetal T4 starts from week 22-24. Severe iodine deficiency was found to be associated with fetal hypothyroidism, mental impairment, and increased neonatal and infant mortality (22). Hynes et al. (23) showed that even mild iodine deficiency had long-term adverse impacts on fetal neurocognition and these adverse effects could be reversed by replacement in childhood. Rydbeck et al. (24) also showed that birth weight and length increased by 9.3 g [95% confidence interval (CI): 2.9-16] and 0.042 cm (95% CI: 0.0066-0.076), respectively, for each 0.1 μg/L increase in maternal UIC (24). Bath et al. (21) also conducted a study known as Avon Longitudinal Study of Parents and Children in 1040 pregnant woman in their first trimester. They compared children’s intelligence quotient (IQ) scores at the age of 8 years and reading ability at 9 years, and they found that children whose mothers had UIC levels less than 150 μg/g in pregnancy had lower IQ scores and reading accuracy (21). Oral et al. (25) showed that 90% of pregnant women were lacking iodine in their pregnancy in İstanbul, which is defined as an iodine-sufficient city and the salt iodization program was not an efficient for the pregnant population. Oguz Kutlu and Kara (19) assessed pregnant women who were in their second trimester in Ankara and they also found that 72.8% of woman had iodine deficiency. Despite mandatory iodized salt use, iodine deficiency can be explained by inconvenient storage or consumption of iodized salt. Erdoğan et al. (18) showed that only 56.5% of people consumed iodinised salt at home, which is lower than the WHO recommedation of 90%, to eradicate iodine deficiency. Anaforoğlu et al. (26) also showed that 64.9% of patients used salt inappropriately in the cooking process and only 25% of patients used proper containers for iodized salt (27). The limitations of this study include the patients’ knowledge about iodized salt and their socioeconomic status. In addition, this was a single center study in the capital city of Turkey, which may not reflect the status of rural areas. In this study, we found that iodine deficiency has not been eradicated despite mandatory iodized salt use, similar to the literature. Iodine status should be assessed in pregnant women, and proper iodine replacement should be advised to patients even before pregnancy. To be successful in the eradication iodine deficiency, from salt iodinization to the cooking process, every step must be checked. One mistake in these steps results in inadequate iodine intake. Iodized salt use education may be given to pregnant women as a part of pregnancy education class. Attention should be given while buying salt, in order to not to take incorrect salt types. Proper iodine replacement should be given to all pregnant woman along with appropriate iodinised salt use.

## Figures and Tables

**Table 1:**
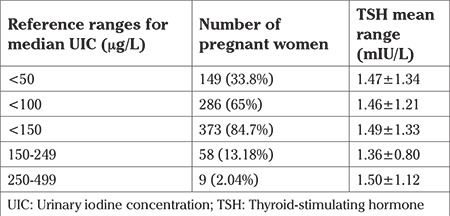
Urinary iodine concentration in pregnant women

**Table 2:**
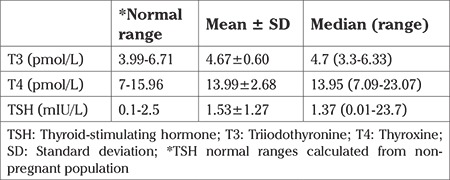
Thyroid values of study population
